# B-Flow and Contrast-Enhanced Ultrasound (CEUS) Features of Subcutaneous Masses and Nodular Lesions in Dogs

**DOI:** 10.3390/vetsci11100516

**Published:** 2024-10-21

**Authors:** Andrea De Bonis, Francesco Simeoni, Andrea Paolini, Martina Rosto, Francesca Del Signore, Laura Bongiovanni, Amanda Bianchi, Roberto Tamburro, Massimo Vignoli

**Affiliations:** 1 Faculty of Veterinary Medicine, University of Teramo, 64100 Teramo, Italy; francsimeoni@gmail.com (F.S.); apaolini@unite.it (A.P.); mrosto@unite.it (M.R.); lbongiovanni@unite.it (L.B.); abianchi@unite.it (A.B.); rtamburro@unite.it (R.T.); mvignoli@unite.it (M.V.); 2 Department of Biomolecular Health Sciences, Faculty of Veterinary Medicine, Utrecht University, 3584CT Utrecht, The Netherlands

**Keywords:** subcutaneous 1, B-flow 2, CEUS 3

## Abstract

Subcutaneous lesions in dogs are common in clinical practice. This prospective clinical study aims to compare B-flow and CEUS for the characterization of subcutaneous lesions evaluating their usefulness to distinguish benign subcutaneous lesions from malignant ones. Dogs were enrolled and ultrasound cine-loops were achieved in B-mode, Colour Doppler, Power Doppler, B-flow and CEUS. Lesions vascularisation highlighted through B-flow and CEUS were classified into five patterns: P1, absence of contrast uptake; P2, enhancement only in the peripheral area of the lesion; P3, thin (<2 mm) and few vessels (<5/field); P4, thicker (>2 mm) and more numerous vessels (>5/field); P5 enhancement with a reticular aspect and both thick and thin bands inside. Patterns highlighted with B-flow and CEUS were compared to a histological diagnosis of subcutaneous lesions. A total of 24 dogs and 30 subcutaneous nodules were included and divided into three groups: 3 non-neoplastic, 16 benign tumours and 11 malignant tumours. There was a statistically significant difference for B-flow and CEUS to differentiate benign tumours from malignant tumours. B-flow and CEUS had an excellent agreement. B-flow and CEUS displayed similar ability to evaluate different patterns and could be helpful in the evaluation of subcutaneous nodules.

## Simple Summary

Subcutaneous lesions are common in dogs. This prospective study aims to compare various ultrasound techniques for investigating subcutaneous lesions and to assess their effectiveness in distinguishing between benign and malignant neoplasia. Dogs were included and ultrasound cine-loops were achieved in B-mode, Colour Doppler, Power Doppler, B-flow and CEUS. Vascularisation highlighted through B-flow and CEUS were classified into five patterns. The vascular patterns obtained with B-flow and CEUS were compared to the histological diagnosis of the subcutaneous lesions. A total of 24 dogs and 30 subcutaneous lesions were included and divided into three groups: non-neoplastic, benign tumours and malignant tumours. CEUS and B-flow were able to help in the differentiation of benign tumours from malignant tumours and achieved an excellent agreement. B-flow and CEUS displayed similar ability to evaluate different patterns and could be helpful in the evaluation of subcutaneous lesions.

## 1. Introduction

Subcutaneous lesions are common in companion animals and daily clinical practice. B-mode ultrasound is the first choice technique to investigate these subcutaneous lesions; however, it cannot differentiate between benign and malignant lesions [1]. The use of Doppler methods, Colour Doppler and Power Doppler, can increase the sensitivity of ultrasound by identifying the vascularisation of the masses. Furthermore, combining different techniques can improve the specificity of the results and, to some extent, predict the malignancy of the masses [2]. A human medicine study suggested that studying vascularisation is useful for defining the characteristics of benign and malignant subcutaneous neoplasms [3].

Doppler is a valuable aid for this investigation and it’s the most common technique used in ultrasound; however, there are numerous limitations regarding Doppler methods for blood flow analysis. The first limitation of Colour Doppler is the dependence on the angle between the beam and the vessel (*beam-vessel angle*): thus, a curved vessel will contain areas without flow. B-flow, on the other hand, is not dependent on the beam-vessel angle and does not present flow ambiguity [4]. Additionally, Colour Doppler presents the artefact of ‘oversaturation’, which causes visualisation of blood flow beyond the vessel boundaries. This can lead to an overestimation of the vessel’s actual size. In contrast, B-flow does not exhibit any vessel overwrite, accurately delineating its boundaries according to its true size. Finally, with Doppler methods, slow flow might not be properly detected, potentially leading to an incorrect diagnosis of vascular occlusion [4].

The B-flow method, a non-Doppler technique developed in the 2000s, allows for the direct and real-time visualisation of blood flow echoes and can be used as a diagnostic aid, addressing some issues of Colour Doppler [4]. B-flow is a digitally encoded ultrasound technology developed by GE Healthcare (Chicago, IL, USA) for visualizing blood flow. It combines coded excitation and tissue equalization, allowing the visualization of moving blood echoes using a grey-scale presentation. This method shows real-time blood movement similar to a conventional angiogram and simultaneously visualizes the surrounding anatomy [5,6]. However, B-flow has limitations that hinder its use in clinical practice. Unlike Colour Doppler, it doesn’t provide information on the speed and direction of flow [7]. For this reason, Colour Doppler ultrasound remains the most used non-invasive technique for visualising blood flow. Nevertheless, B-flow is used as a complementary method and is a valuable tool for further studying vascularisation [7,8]. To the author’s knowledge, there are no publications in veterinary literature regarding the use of B-flow in the study of subcutaneous masses. The few studies of B-flow in veterinary medicine are mostly of hepatic and renal vascularisation and characterise hepatic neoplastic lesions [9] and testicular tumours in dogs [10,11]. In human medicine, the clinical application of B-flow has been used at the beginning for the vascularisation of the carotid artery [12] and is now mainly used in the evaluation of renal transplants, thyroid neoplasia and vascular medicine [6].

The contrast-enhanced ultrasound (CEUS) is another valuable tool to study vascularisation. The second-generation contrast agent used is a solution consisting of gas-filled microbubbles of sulphur hexafluoride, detectable by ultrasound and, when injected into the bloodstream, has an exclusively vascular localization [13]. This technique allows for the evaluation of thinner vessels (40 μm) compared to Doppler and B-flow, and it’s useful to confirm the absence of vascularisation. Also allows to characterize the lesion vascularisation [14]. CEUS can be administered safely without the need to investigate renal function beforehand, as it is excreted via the respiratory route and does not contain iodine [15]. Finally, it’s able to assess a dynamic enhancement of lesions and tissue like the CT and MRI, in contrast with the traditional US techniques, providing the advantages of real-time imaging [13]. In veterinary medicine, studies using CEUS in the evaluation of subcutaneous masses are lacking; there are few human medicine studies about CEUS in musculoskeletal soft tissue masses [1]. In these studies, the perfusion patterns found were used as tools to differentiate between malignant and benign tumours [1]. In another human medicine study, CEUS was used to evaluate subcutaneous soft tissue tumours and the vascularisation masses was divided into different perfusion patterns [3].

This prospective study aims to compare different imaging techniques, particularly B-flow ultrasound and CEUS, to identify the most useful method for distinguishing malignant from benign lesions in subcutaneous tissue. To date, to the author’s knowledge, no studies in veterinary medicine have investigated the vascularisation of subcutaneous lesions using B-flow and CEUS, comparing these two techniques. This study hypothesises that certain vascular patterns may aid in the classification of subcutaneous neoplasms, particularly in distinguishing whether a neoplasm is benign or malignant. The second aim of the study is to evaluate the agreement between the two imaging modalities, and we hypothesize that B-flow would achieve similar results compared to CEUS.

## 2. Materials and Methods

In this prospective study, dogs with subcutaneous masses or nodular lesions presented to the imaging diagnostics unit of the University Veterinary Teaching Hospital (OVUD) at the Department of Veterinary Medicine, University of Teramo, from November 2021 to June 2024, were included. Sex, breed, age of the dogs and the dimensions and location of the subcutaneous lesions were recorded. The dogs underwent a complete physical exam and were subsequently brought to the OVUD for ultrasound and histopathological examination of the lesion. Colour and Power Doppler, B-flow, and CEUS were compared to evaluate the vascularisation characteristics of subcutaneous lesions. The vascular patterns identified with these methods were then compared to the histological diagnosis of the subcutaneous lesions obtained through biopsy. Ultrasound examinations and biopsies were performed in dogs under sedation. Before sedation, all dogs underwent a pre-anaesthetic evaluation by the anaesthetist in the facility, which included an assessment of each animal’s cardio-respiratory function and haematological-biochemical tests. Sedation was administered by adapting the anaesthetic protocol according to the patient’s temperament and individual anaesthetic risk; the dogs were monitored throughout the entire procedure, and vital parameters were recorded every 5 min on a dedicated chart. The patient’s recovery was monitored until they fully regained consciousness. The ultrasound examinations of the subcutaneous lesions with cine-loops and static images and the tissue sampling for histological investigation were performed by a resident of the European College of Veterinary Diagnostic Imaging (ECVDI) (ADB) under the supervision of an ECVDI diplomate (MV). The skin area above the subcutaneous lesions was clipped and aseptically prepared. The cine-loops and static images were standardised and obtained in the widest section of the lesions on the sagittal plane.

The inclusion criteria for the study were as follows: patients with at least one subcutaneous lesion, ultrasound cine-loops and static images for each mass, using B-mode, Colour Doppler, Power Doppler, B-flow, and CEUS techniques and histopathological evaluation of the mass. The study excluded dogs with purely epithelial neoplasms or those crossing the deep muscle fascia. Patients without any diagnostic techniques or histological diagnosis were also excluded. All owners were informed about the experimental procedure, data collection for experimental purposes, and related anaesthetic risks. Owners signed an informed consent for all procedures performed. All procedures were conducted under national animal welfare regulations (Legislative Decree No. 26, 4 March 2014), and the study was approved by the Ethical Committee of the Department of Veterinary Medicine at the University of Teramo. Prot. n. 27086 of the 27 October 2021 (2021-UNTECLE-0027086).

### 2.1. B-Mode Ultrasonography

The ultrasound machine used for the study is a General Electric Logiq S8. The ultrasound examination was performed with a linear probe L11 with a frequency range from 9 MHz to 12 MHz. B-mode ultrasound was used to assess the following characteristics of subcutaneous masses and nodular lesions: the shape, margins (regular/irregular; well-defined or irregular poorly defined), dimension, echotexture (homogeneous, heterogeneous), echogenicity compared to surrounding tissues (isoechoic, hypoechoic, hyperechoic, anechoic), and the presence of any calcifications.

### 2.2. Colour Doppler, Power Doppler, B-Flow e CEUS

Vascularisation of the subcutaneous lesions was assessed using Colour Doppler, Power Doppler, B-flow, and CEUS techniques. The vascularisation visualised with Colour and Power Doppler was classified as (a) absent; (b) mild; (c) moderate; and (d) intense. Colour and Power Doppler settings were constant for each exam: medium wall filter, Colour gain of 70% and pulse repetition frequency (PRF) ranging from 0.75 kHz to 1.4 kHz to detect slow blood flows [16].

**Figure 1:**
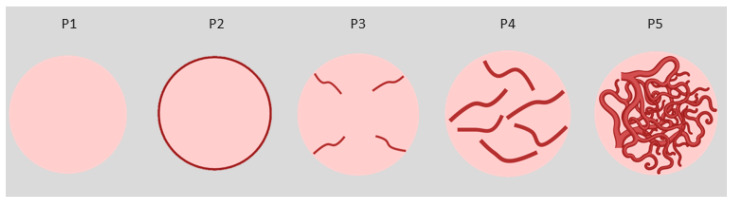
Five perfusion patterns were observed in subcutaneous masses and nodular lesions based on the distribution and morphology of the vessels. Pattern 1; Pattern 2; Pattern 3; Pattern 4; Pattern 5.

B-flow and CEUS methods identified different vascularisation patterns in a 2015 study that analysed subcutaneous nodular lesions in human medicine [1]. The different patterns visualised in the De Marchi study were used for the present study (Figure 1). The patterns considered are as follows:

P1: Absence of vessels (B-flow) or contrast uptake (CEUS);
P2: Presence of vessels (B-flow) or contrast uptake only in the peripheral area of the lesion (CEUS);
P3: Presence of vessels (B-flow) or contrast uptake (CEUS) in thin (<2 mm) and few vessels (<5/field);
P4: Presence of vessels (B-flow) or contrast uptake (CEUS) in thicker (>2 mm) and more numerous vessels (>5 per field);
P5: Presence of numerous vessels (B-flow) or contrast uptake (CEUS) with a reticular aspect and both thick and thin bands inside.

The contrast agent for CEUS evaluation (Sonovue^®^, Bracco Imaging Italia srl, Milano, Italia) was administered through an intravenous catheter inserted into a cephalic vein, with two boluses given at a dose of 0.03 mL/kg, immediately followed by a saline flush (0.9% NaCl) of 1 mL after each bolus [17]. The time was recorded from the contrast agent administration for 90 seconds. The corresponding vascularisation pattern for each mass and nodular lesion was evaluated at its maximum enhancement [3] and through a qualitative evaluation a pattern was assigned. Cine-loops and static images were jointly evaluated by an ECVDI resident (ADB) and an ECVDI diplomate (MV) to reach an agreement on the vascularisation pattern of each subcutaneous nodular lesion and mass.

The different vascular patterns were then compared with the histological diagnosis. Based on histological diagnoses, the nodular lesions were finally divided into 3 subgroups: (a) Non-neoplastic; (b) Benign neoplasia; (c) Malignant neoplasia. The different types of vascularisation patterns obtained with CEUS and B-flow were compared to evaluate the agreement between the two modalities.

### 2.3. Histological Diagnosis

All the subcutaneous masses and nodular lesions underwent histopathological examination, and samples were collected using an ultrasound-guided semi-automatic Tru-cut biopsy needle or with a punch biopsy. Before the biopsy, the area was disinfected with a povidone-iodine soapy solution followed by ethanol. A small incision was made with a scalpel blade on the surface of the masses, through which the Tru-cut needle (14 G) was inserted. The sample was collected with a small needle (23 G) and preserved in appropriate containers with 10% formalin. Haematoxylin and eosin (HE)-stained slides were obtained from FFPE (formalin fixed paraffin embedded) tissues and examined by a pathologist certified by the European College of Veterinary Pathologists (ECVP) (LB).

### 2.4. Statistical Analysis

The statistical analysis was performed using the *Jamovi* software (Version 2.3). The differences in the distribution of the parameters considered in relation to the different groups were analysed using a one-way analysis of variance (ANOVA) for normally distributed data or with the Kruskal-Wallis test for non-normally distributed data. Dwass-Steel-Critchlow-Fligner pairwise comparisons and the B-flow & CEUS Interrater Reliability were performed, and then a Cohen’s Kappa and a *p* value of each parameter were calculated. Kappa values were interpreted as follows: between 0.21 and 0.40 were considered as slight agreement, between 0.41 and 0.60 as moderate agreement, while values between 0.61 and 0.80 as substantial agreement and values over 0.80 as excellent agreement. A *p* value < 0.05 is considered statistically significant for each test.

## 3. Results

The study included 24 dogs with a total of 30 subcutaneous lesions. Of these, 16 were mixed breeds, 4 were Bracco Italiano, 1 Dogo Argentino, 1 Maremma Sheepdog, 1 Labrador Retriever, and 1 Toy Poodle. Age ranged from 1 to 18 years (average age 10.1 years), and weights ranged from 4.5 kg to 38 kg. Regarding sex, there were six intact males, eight neutered males, and ten spayed females. Of the 24 dogs included in the study, 4 had more than one subcutaneous lesion. Of the 4 dogs with multiple subcutaneous lesions 2 patients had 2 lesions each (one had 2 haemangiomas and the other 2 lipomas) and 2 patients had 3 lesions each (one had 1 soft tissue sarcoma and 2 lipomas and the other had 3 mast cell tumours). The subcutaneous lesions varied in size, ranging from 0.7–18 cm in length × 0.5–12 cm in height. The locations of the lesions were: thoracic wall 12, abdominal wall 7, shoulder 4, inguinal region 2, perianal region 1, preputium 1, neck 1, head 1, iliac wing 1.

### 3.1. B-Mode Ultrasonography

Using B-mode ultrasound, 23 subcutaneous lesions had an ovoid shape (9 lipomas, 4 mast cell tumours, 3 haemangiomas, 3 soft tissue sarcomas, 3 inflammatory granulomas, 1 adenocarcinoma of the apocrine gland), 3 were irregular (3 soft tissue sarcomas), and 4 were round (4 lipomas). The margins were well-defined in 17 lesions, while in 13 they were poorly defined. Out of the 17 well-defined marginated lesions: 6 were lipomas, 4 were mast cell tumours, 3 were soft tissue sarcoma, 3 were haemangiomas, and 1 was inflammatory granuloma. Regarding the 13 poorly defined marginated lesions: 7 were lipomas, 3 were soft tissue sarcoma, 2 were inflammatory granulomas, and 1 was adenocarcinoma of the apocrine gland. The echotexture was heterogeneous in 25 lesions and homogeneous in 5. The 5 homogenous lesions were all lipomas, while the remaining lesions were heterogenous. Regarding echogenicity, 17 were predominantly hypoechoic (5 lipomas, 3 soft tissue sarcoma, 3 mast cell tumours, 3 inflammatory granulomas, 3 haemangiomas), 8 were isoechoic (7 lipomas and 1 mast cell tumour), and 5 were hyperechoic with hypoechoic portions (3 soft tissue sarcomas, 1 adenocarcinoma of the apocrine gland, 1 lipoma). The only lesion that presented mineralisation was a soft tissue sarcoma.

### 3.2. Colour and Power Doppler

The data obtained on vascularisation analysed using Colour and Power Doppler were comparable. In 13 subcutaneous lesions, no vascularisation was detected; in 8 there was mild vascularisation, in 6 moderate and in 3 the vascularisation was intense. The data are summarized in Table 1.

### 3.3. B-Flow

**Figure 2:**
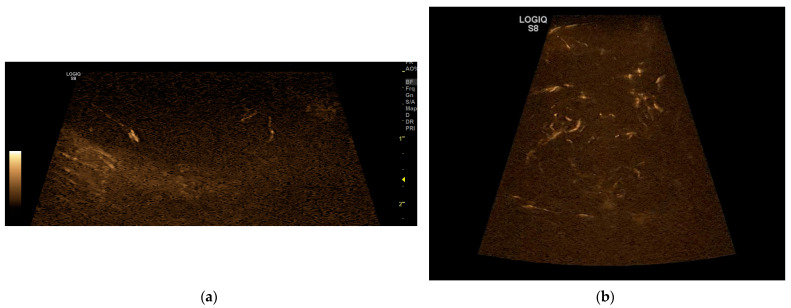
(**a**) Pattern 3 of a Haemangioma obtained with B-flow; (**b**) Pattern 5 of a Soft Tissue Sarcoma with B-flow.

On the B-flow evaluation, all five patterns were detected. In 14 dogs, a P1 pattern was found; in 2 dogs, a P2 pattern; in 5 dogs, a P3 pattern; in 5 dogs, a P4 pattern; and in 4 dogs a P5 pattern (Figure 2). In a dog, there was the twinkling sign due to mineralisation. These data are resumed in the table below compared to the data obtained with CEUS (Table 2).

### 3.4. CEUS

**Figure 3:**
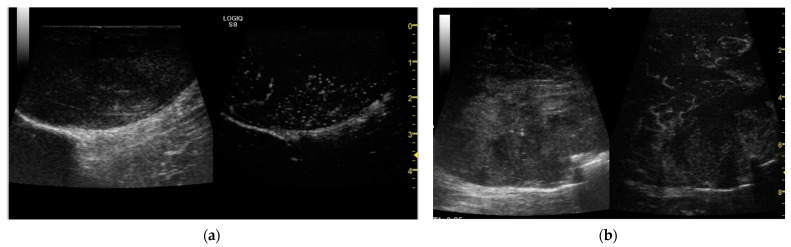
(**a**) Pattern 4 of a Mast cell tumour obtained with CEUS; (**b**) Pattern 5 of a Soft Tissue Sarcoma with CEUS.

After the administration of the contrast agent, the qualitative evaluation revealed the following patterns: in 11 dogs the P1 pattern was detected; in 2 dogs the P2; in 8 dogs the P3; in 5 dogs the P4; and in 4 dogs the P5 (Figure 3). These data are resumed in Table 2 compared to the B-flow.

### 3.5. Statistical Analysis

The subcutaneous masses and nodular lesions were then divided into 3 subgroups: non-neoplastic, benign tumours and malignant tumours; were observed 3 non-neoplastic lesions (3 inflammatory granulomas), 16 benign tumours (13 lipomas, 3 haemangiomas) and 11 malignant tumours (6 soft tissue sarcomas, 4 mast cell tumours, 1 adenocarcinoma of the apocrine gland). The frequencies of B-flow and CEUS patterns were then calculated for each group and resumed in the table below Table 3.

Pairwise comparison tests were executed between benign and malignant neoplasia. Given the small number of cases in the non-neoplastic group, they were not included in the statistical analysis. The *p* value was <0.05 when comparing benign and malignant tumours in CEUS or B-flow, respectively 0.016 and 0.002. The results are illustrated in Table 4.

Lastly, the Interrater Reliability was calculated for these two techniques. A Cohen’s Kappa of 0.819 was obtained, with an agreement of 86.7%. The *p* value was <0.001.

## 4. Discussion

The use of CEUS and B-flow in the evaluation of subcutaneous masses and nodular lesions can be a valuable aid in differentiating between benign and malignant tumours as the results of this study have made it possible to associate detected patterns with malignancy. However, this does not eliminate the need for a histological examination.

B-mode did not yield significant information regarding the differentiation between benign and malignant subcutaneous lesions. No association was found between the shape and margins of the tumour and malignancy: most of the subcutaneous lesions, both benign and malignant, were oval in shape, and the margins in malignant lesions could be either well-defined or poorly defined.

Regarding the heterogeneity of the echostructure, most benign neoplasia, including lipomas and haemangiomas, had a heterogeneous echostructure. However, some lipomas presented a homogeneous structure, which is inconsistent with the literature where lipomas are described as having a heterogeneous echo structure [18] or a striped appearance [19]. Malignant tumours, on the other hand, exhibited mainly heterogeneous echostructures.

This study did not find an association regarding the different types of echogenicity, as the subcutaneous lesions were both hyperechoic and hypoechoic compared to the surrounding fat. The literature showed conflicting data: in one study, hyperechogenicity of the subcutaneous lesion was associated with soft tissue sarcomas [20], while in another study, hyperechogenicity was associated with lipomas [18]. Another study highlighted an association between hypoechogenicity and malignancy [21], while a subsequent study found no relevant association, attributing it to different pathological processes involved, such as the presence of tissue necrosis and inflammation [2]. Furthermore, a study conducted on canine lipomas found that echogenicity can vary and that there is no uniformity in the findings; this variation can depend on their cellularity and the amount of fat they contain [19]. This was also observed in this study, as the lipomas were either hypoechoic or isoechoic compared to the surrounding fat.

Regarding the Doppler techniques, Colour Doppler and Power Doppler displayed the same vascularisation patterns. This is in partial disagreement with previous studies in the literature, which highlighted the greater capability of Power Doppler to detect low flow and lower velocity, typical of neoplasms, compared to Colour Doppler [22]. This finding contrasts also with a more recent study conducted on splenic masses, where it was found that vascularisation appeared more pronounced on Power Doppler compared to Colour Doppler [23]. In other veterinary studies, Power and Colour Doppler achieved almost the same results evaluating the vascularisations of hepatic masses and mammary neoplasia [24,25]. Furthermore, in another study of testicular neoplasia, Power and Colour Doppler had an excellent agreement [10].

In this study, most benign neoplasia exhibited an absence of vascularisation. In some benign tumours slight vascularisation was detected, both peripheral and intralesional, but always with a small number of vessels. In contrast, malignant neoplasms mostly showed moderate vascularisation with both intralesional and peripheral signals, and in 3 cases, the vascularisation was intense. Soft tissue sarcomas exhibited moderate to intense vascularisation, with multiple peripherals and intralesional vessels, while mast cell tumours showed moderate or mild vascularisation. However, in the case of a cavernous haemangioma, vascularisation appeared moderate with both central and peripheral signals. This does not completely discriminate between benign and malignant neoplasms, as benign neoplasms can also exhibit increased vascularisation, and mast cell tumours in this study predominantly had mild to moderate vascularisation. The vascularisation types were overlapping between the different groups and a clear correlation would not have been possible to show. Almost all lipomas, excluding one lipoma with a mild vascularisation, showed an absence of Doppler signals. This suggests that an absence of vascularisation, as detected by Doppler techniques, could be associated with a benign neoplasia such as lipoma. However, previous studies have mentioned that the lack of vascularisation detected by Colour Doppler is not indicative of its actual absence, as it has a poor ability to detect slow flows [2]. This is supported by another previous study, which found that the absence of vascularisation can be observed in both benign masses with small-calibre vessels and/or lesions with arterial pressures too low to be detected [10] and in malignant masses that have undergone total necrosis [26].

Regarding the use of B-flow in the evaluation of subcutaneous lesions, to the author’s knowledge, there are no studies in either human or veterinary medicine that delve into this topic. B-flow has been applied to various types of tumours in human medicine, like liver tumours [9] and thyroid nodules [27] and compared with Doppler methods to assess the sensitivity of the two techniques in evaluating tumour vascularisation. Some studies have shown that the sensitivity in detecting vascularisation is comparable between Colour Doppler and B-flow [9]. However, it has been noted that B-flow is more specific regarding the true size of the vessels [28].

In this study, the patterns observed with B-flow were related to those detected with CEUS, using the same classification to standardise the patterns obtained and achieve uniform results. In the present study, most benign neoplasms exhibited an absence of vascularisation (P1), all the lipomas showed this pattern, except in one case with B-flow and in 3 cases with CEUS where a P3 pattern was present. However, two haemangiomas, although benign neoplasia, showed P3 patterns with multiple intralesional vessels. Additionally, 3 soft tissue sarcomas, the adenocarcinoma of the apocrine glands and a mast cell tumour (only with CEUS) showed the presence of numerous vessels with reticular aspect (P5), while another soft tissue sarcoma along with 4 mast cell tumours presented numerous intralesional vessels > 2 mm (P4). One of the soft tissue sarcomas showed few vessels per field < 2 mm (P3).

The patterns detected with the qualitative analysis of CEUS images overlap with those observed with B-flow. However, in two lipomas, the presence of vascularisation with a P3 pattern was detected, while a soft tissue sarcoma was classified as P4 instead of P5 and a mast cell tumour was classified as P5 instead of P4. This method allowed the detection of small vessels that were otherwise invisible with B-flow and Doppler methods, likely due to the CEUS’s ability to detect vessels as small as 40 µm [13].

The Kappa value obtained between B-flow and CEUS was 0.819, which indicates an excellent agreement between these two techniques. The agreement obtained was 86.7% and the *p* value was <0.001, so CEUS and B-flow have almost the same ability to detect different vascular patterns in subcutaneous lesions.

In this study, either with B-flow or CEUS, most benign lesions showed the P1 pattern, except for three lipomas and two haemangiomas, which showed the P3 pattern. This is consistent with a previous paper where the absence of vascularisation was linked to benign lesions [29].

The P2 pattern was observed only in non-neoplastic subcutaneous lesions, according to the literature [1]. However, one previous study claims that this pattern could be associated with malignant tumours due to the decreased blood supply and a higher central interstitial pressure than in benign masses, which results in central necrosis with no contrast enhancement [29]. Unfortunately, the data regarding this pattern are lacking and, in this study, only two subcutaneous lesions presented this pattern, so further studies are necessary.

The P3 pattern was mostly associated with benign lesions, including lipomas and haemangiomas. However, two soft tissue sarcoma also exhibited this pattern, making associating it with a specific category difficult. In other studies, it was not possible to define this pattern properly because there were no statistical differences between benign and malignant tumours and this pattern can be present in both groups [1,3]. However, in one study it was defined as a “low risk” of malignancy for musculoskeletal tumours [1].

In this study, the P4 and P5 patterns were found only in malignant neoplasia.

The majority of P5 patterns found in the study were in soft tissue sarcomas. This finding is consistent with a previous study that had already associated this pattern with sarcomas [29]. This result was predictable, and it could aid in the classification of a neoplastic lesion that presents this pattern with ultrasonography. Although, a Mast cell tumour and the adenocarcinoma of the apocrine gland were classified as P5, a correlation between vascularisation pattern and histopathological diagnosis cannot be achieved.

Based on this data, the *p* value obtained was <0.05 when comparing benign and malignant tumours in CEUS or B-flow respectively 0.016 and 0.002 reaching a statistical significance. These results disagree with a previous study in human medicine regarding the CEUS applied to subcutaneous tumours, in which no differences were observed between benign and malignant tumours [3]. In this study 7 patterns were described, gathering patterns 6 and 7 as potentially malignant and patterns 1 to 5 as potentially benign lesions. However, they did not yield statistically significant differences between benign and malignant tumours [3]. In a recent study in veterinary medicine, superficial neoplasia were evaluated through CEUS with several overlapping features between benign and malignant neoplasia [30].

In this study, the results obtained showed that B-flow and CEUS could help differentiate benign and malignant tumours with a *p* value < 0.05. There are some limitations in this study. Most of the subcutaneous lesions were benign neoplasms. Additionally, among the benign neoplasms, lipomas were overrepresented compared to other tumours. Another limitation is the small number of cases included, as improving the reliability of the results would require further investigation with additional cases. Another potential limitation would have been that the ECVDI resident and the ECVDI diplomate evaluated the same subcutaneous lesion with different techniques in sequences and were not blinded. Therefore, although it is not possible to determine a definitive correlation between the study of vascularisation and the benign or malignant nature of the subcutaneous lesions, some patterns can aid in differentiation, and thus, this study can be considered for future investigations.

Ultrasonography is a rapid and non-invasive diagnostic method for examining subcutaneous masses and nodular lesions in dogs. The application of various techniques, particularly Doppler methods, B-flow, and CEUS can serve as complementary tools in the evaluation of subcutaneous lesions. As highlighted in previous studies, Doppler techniques seem to have a certain accuracy in distinguishing between benign and malignant tumours: they have shown that benign formations are hypovascularized, while hypervascularisation is primarily associated with malignant tumours.

CEUS and B-flow were applied for the first time in the study of subcutaneous lesions in dogs, and both techniques provided comparable results with a Kappa value of 0.819 and an excellent agreement. Therefore, either of these techniques can be used as valid tools for assessing the vascularisation of subcutaneous lesions and to help in the differentiation between benign and malignant tumours.

Given the same ability to evaluate the vascularisation of subcutaneous lesions, B-flow is a valid alternative to CEUS for this evaluation; although theoretically, CEUS can visualise even smaller vessels [31] compared with B-flow, the latter offers certain advantages. B-flow can be used when the contrast agent is not available or due to economic concerns. Additionally, it can be performed to avoid the insertion of a venous catheter, which is necessary for the administration of the contrast agent. Another advantage is that this method is already available in some ultrasound machines and can be applied in the same manner as Doppler techniques. Consequently, B-flow is an inexpensive, non-invasive and simple tool to characterize the vascularisation of subcutaneous lesions.

Although the results obtained are promising, cytological and or histopathological examination remains the gold standard for a definitive diagnosis of the subcutaneous lesion.

While the ultrasound examination is useful, it does not play a primary role in the diagnosis of subcutaneous lesions. However, it is useful for the real-time assessment of the vascularisation of the subcutaneous lesions as a complementary tool. This can aid in distinguishing between benign and malignant neoplasia, providing a general overview before the histological diagnosis.

## Figures and Tables

**Table 1:** Summary of the results obtained through Colour and Power Doppler.

Diagnosis	N°	Colour Doppler				Power Doppler			
		A	Mi	Mo	I	A	Mi	Mo	I
Lipoma	13	12	1	-	-	12	1	-	-
Haemangioma	3	-	3	-	-	-	3	-	-
Infla. Granuloma	3	1	1	1	-	1	1	1	-
Soft Tissue Sarcoma	6	-	1	2	3	-	1	2	3
Mast Cell Tumour	4	-	2	2	-	-	2	2	-
Adenocarcinoma	1	-	-	1	-	-	-	1	-

A: absent; Mi: mild; Mo: moderate; I: intense.

**Table 2:** Summary of results obtained through B-flow and CEUS, compared to the histological diagnosis.

Diagnosis	N°	B-Flow					CEUS				
		P1	P2	P3	P4	P5	P1	P2	P3	P4	P5
Lipoma	13	12	-	1	-	-	9	-	4	-	-
Haemangioma	3	1	-	2	-	-	1	-	2	-	-
Infla. Granuloma	3	1	2	-	-	-	1	2	-	-	-
Soft Tissue Sarcoma	6	-	-	2	1	3	-	-	2	2	2
Mast Cell Tumour	4	-	-	-	4	-	-	-	-	3	1
Adenocarcinoma	1	-	-	-	-	1	-	-	-	-	1

**Table 3:** Frequencies of the patterns obtained with CEUS and B-Flow were divided for each group.

**B-Flow**			
**Pattern**	**Non-Neoplastic**	**Benign Tumour**	**Malignant Tumour**
P1	1	13	
P2	2		
P3		3	2
P4			5
P5			4
**CEUS**			
**Pattern**	**Non-Neoplastic**	**Benign Tumour**	**Malignant Tumour**
P1	1	10	
P2	2		
P3		6	2
P4			5
P5			4

**Table 4:** Statistical analysis CEUS and B-Flow, divided for each group.

**B-Flow**		***p* Value**
Benign Tumour	Malignant Tumour	0.002
**CEUS**		***p* Value**
Benign Tumour	Malignant Tumour	0.016

## Data Availability

The datasets presented in this article are not readily available due to privacy reasons.
